# Fears and Worries at Nighttime in Young Children: Development and Psychometric Validation of a Parent-Report Measure (FAWN-YC)

**DOI:** 10.1007/s10578-024-01758-3

**Published:** 2024-09-16

**Authors:** Amy Shiels, Laura Uhlmann, Lara J. Farrell, Erinn Munro-Lee, Caroline L. Donovan

**Affiliations:** 1 School of Applied Psychology, 176 Messines Ridge Rd, Mount Gravatt, Brisbane, QLD 4122 Australia; 2 Griffith University Centre for Mental Health, Mount Gravatt, Australia

**Keywords:** Preschoolers, Nighttime fears, Dark, Measure, Parent-report

## Abstract

This paper outlines the development and psychometric evaluation of the Fears and Worries at Nighttime—Young Children (FAWN-YC) scale; a parent-rated measure for children aged 3–5 years. Based on previous literature, it was hypothesised that the measure would be represented by a six-factor solution, with four clusters of fear types and two behavioural manifestations of fears. Exploratory factor analysis (EFA; N = 436) and confirmatory factor analysis (CFA; N = 383), resulted in a final 17 items that loaded onto 3 factors: Nighttime Fear Focus (8 items, α = 0.92), Bedtime/Sleep Avoidance and Interference (5 items, α = 0.90), and Dark Fear (4 items, α = 0.88). Evidence of convergent validity was found through strong associations between the total score and subscales of the FAWN-YC with measures of child anxiety, fear, sleep, externalizing and conduct problems. Furthermore, there was support for divergent validity (through a very weak to no relationship with a measure of prosocial behaviours), and evidence for temporal stability was also established with 2-week test–retest reliability. Overall, the results provide strong preliminary evidence for the reliability and validity of the FAWN-YC total score and subscales. Implications for the use of the measure in research and clinical practice are discussed.

**Supplementary Information:**

The online version contains supplementary material available at 10.1007/s10578-024-01758-3.

## Introduction

Nighttime fears are a heterogenous group of fears that include separation fears, personal safety fears, imagination-based fears, and darkness fears [1–4]. Nighttime fears are particularly prevalent in young children and are often considered developmentally normal, with almost 60% of 4- to 6-year-olds experiencing difficulties with fear at night [4]. Although nighttime fears are transient for many children, approximately 10 to 30% experience fear at night that is severe, persistent, interferes substantially with sleep, and requires significant family accommodation [4–7]. Cross-sectional literature indicates that compared to controls, preschool aged children with severe nighttime fears demonstrate increased general fears, internalising and externalising behaviours, and lower effortful control [8]. Similarly, El Rafihi-Ferreira et al. [9] found that internalising behaviours were positively associated with parent-reported child nighttime fear in a sample of preschool aged children whose parents attended a nighttime fear treatment program.

Indeed, severe nighttime fears are impairing, reaching diagnostic thresholds for an anxiety disorder (i.e., specific phobia and/or separation anxiety), and/or a sleep disorder (most commonly insomnia [10, 11]. Anxiety and behavioural sleep disorders in the preschool developmental period can lead to numerous problematic consequences in both the short- and long-term. Anxiety in the preschool years frequently endures into later childhood and beyond, and predicts lower school engagement, poorer peer relations and functional impairment during the school-age years, as well as sleep difficulties and psychopathology into adulthood [4, 12, 13]. Similarly, behavioural sleep problems in the preschool period have been shown to persist into adolescence if untreated, with numerous deleterious social, emotional and educational consequences [14–20].

Nighttime fears have recently gained renewed attention in the paediatric sleep literature as an important contributor to behavioural insomnia symptoms during the preschool developmental period [12, 21]. In a recently published 25-year review of nighttime fears in children, Lewis et al. [21] analysed studies employing behavioural, and cognitive-behavioural interventions. It was concluded that treating nighttime fears resulted not only in significant reductions in nighttime fears and dark phobias, but also significantly improved sleep and reduced general fears, anxiety, internalising and externalising behaviour problems in children aged 3–12 years. Thus, for many young children, nighttime fears are at the root of difficulties with sleep and problems at bedtime. Given the high prevalence and deleterious consequences associated with nighttime fears, it is crucial that we comprehensively understand them and have a measure to screen for them early in life.

Nighttime fears vary both in terms of their focus and the behavioural difficulties resulting from them. With respect to focus, some children experience one specific fear, while others experience multiple fears, with nighttime fears generally clustering into presentations of separation fears (being away from parents), darkness-related fears (sounds, shadows), personal safety fears (being harmed by an intruder) and fears of the imagination (e.g., ghosts; [1–4]). With respect to behavioural difficulties manifesting as a result of nighttime fears, children may demonstrate resistance and refusal to participate in activities leading up to bedtime, they may cry and call out at bedtime, they may be unable to stay in a darkened bedroom or leave their room for other reasons [9, 22–25].

In order to effectively treat nighttime fears, clinicians must consider both the *type* of nighttime fear and the *behavioural outcomes* they produce. For example, a child who cries and repeatedly comes out of their room because they are afraid of the dark and therefore cannot remain alone in their darkened bedroom, will require a specific intervention. The treatment plan would include an exposure hierarchy which gradually exposes the child to being comfortable in the dark. This intervention would also include parenting strategies such as parent-mediated child relaxation skills, and effective praise. Alternatively, a young child who tantrums and refuses to take part in the bedtime routine because they are worried about being separated from their family at bedtime will require an alternative intervention. This treatment plan would likely begin with parenting strategies such as behaviour management skills (i.e., a bedtime routine reward chart, effective praise, how to negotiate with a young child, etc.) followed by an exposure hierarchy focused on gradually separating from their caregiver at night [6]. A comprehensive measure of nighttime fears and the behavioural outcomes they lead to, may assist in the development of an individual case formulation and evidence-based treatment plan. However, to date, such a measure has not been developed.

Despite nighttime fears being an important factor in the development and maintenance of sleep problems in young children, their assessment is notably absent from the vast majority of both paediatric anxiety and sleep measures developed to date. In fact, a validated measure for this vulnerable, preschool aged developmental period (i.e., ages 3–5 years) is yet to be developed. Within the paediatric *anxiety* literature, there are a few psychometrically validated measures assessing dark or night fears, however there is currently no measure for use in preschool aged populations. For instance, the self-report Fear of the Dark Scale [26], was designed for use with adults and adolescents. The child self-report Nighttime Fears Scale [27] and the child self-report and parent-report Children’s Nighttime Fear Survey [3] were developed for children over 7 and 8 years of age respectively. Turning to the paediatric *sleep* literature there exists only two measures that have been validated with a population which includes preschool aged children that include *aspects* of nighttime fears: the parent-report Children’s Sleep Habits Questionnaire [28] and the parent-report Manifestations and Vulnerabilities of Behavioural Insomnia in Childhood Scale [29, 30]. However, both of these are comprehensive *sleep* measures with minimal items pertaining to fears and anxiety at night. For clinical and research purposes, both sleep measures provide insufficient detail on the *type* of nighttime fears and the particular *behavioural manifestations* that may result from them.

Both self-report and parent-report rating scales are limited by the amount and type of information that can be collected. Additionally, when it comes to parent-reports, Muris et al.’s [4] study of 4- to 12-year-olds concluded that parents provided a remarkable underestimation of the frequency of their child’s nighttime fears. Indeed, when examining the results reported separately by age group (4–6 years, 7–9 years and 10–12 years), there was a vast difference between child and parent reported fear frequency in the two older age groups. However, in the youngest group, there was a much smaller difference between the percentage of children (58.8%) and their parent (44.3%) reporting nighttime fears (operationalised in Muris et al. [31] as frequency). As for nighttime fear *content* in childhood, studies have found that children and their parents report very similar results [3, 4]. Most scales assessing anxiety and sleep in preschool aged children are parent-report [32, 33]. Designing a parent-report measure of nighttime fears allows for clinicians and researchers to easily include the measure in a relevant survey battery. Furthermore, when comparing parent-report measures with other methods of data collection for dark and nighttime fears (e.g., interviews, tests of passive approach, psychophysiological records, see Orgilés et al. [27], psychometrically validated parent-report scales offer a reliable, standardised, time efficient and cost-effective method to collect data on young children.

Given the lack of a validated measure of nighttime fears in preschoolers, researchers examining the treatment of nighttime fears in young children have instead relied on adapted interviews [8, 25, 34, 35], checklists [2], modifications of other valid measures (such as general fear measures), or unvalidated measures [36–38]. A psychometrically validated parent-report measure of nighttime fears for children in the preschool developmental period is therefore warranted for both research and clinical purposes.

The aim of this research was to develop and psychometrically validate, a parent-rated measure of nighttime fears in preschool aged children titled Fears and Worries at Nighttime – Young Children (FAWN-YC). Based on previous research [1, 2, 4] it was hypothesised that the FAWN-YC would best be explained by a 6-factor solution including four nighttime fear clusters (1) personal safety fears, (2) separation fears, (3) imaginal/fantasy fears, and (4) inherent characteristics of the dark fears), and two avoidance and interference clusters (5) at bedtime/sleep and (6) in the dark/at nighttime). It was also hypothesised that these factors would be correlated with each other and may represent a general factor structure of overall nighttime fears and worries.

It was further hypothesised that scores on the FAWN-YC would demonstrate strong internal consistency, strong convergent validity (i.e., positive correlations) with theoretically related constructs including measures of fear, anxiety, sleep problems, sleep anxiety, conduct problems and emotional problems, as well as divergent validity (i.e., low, weak to no relationship) with the theoretically unrelated construct of prosocial behaviour. Finally, it was hypothesised that the test–retest reliability of the FAWN-YC would be strong over a 2-week period.

## General Method

Godfred et al.’s [39] three phase approach for scale development in health, social, and behavioural sciences was used to guide scale development in conjunction with Spruyt and Gozal’s [40] steps in paediatric sleep tool development. The three phases include: Phase (1) *Item development*; aimed to generate items and assess the measure content through use of an expert panel. Phase (2) *Scale development*; aimed to pilot test the measure, conduct item reduction analyses and conduct an Exploratory Factor Analysis (EFA) to examine the factor structure. Phase (3) *Scale evaluation*; aimed to confirm the factor structure using Confirmatory Factor Analysis (CFA) and examine the psychometric properties of validity (convergent validity and divergent validity) and reliability (internal consistency and test–retest reliability).

### Procedure

For all phases, participants (i.e., parents of children aged 3–5 years) were recruited internally at the university through a staff and student call for research and the first-year psychology research pool, and externally through advertisement on social media and in private childcare centres, primary schools and early childhood groups and associations. Data for all phases were collected online using Lime Survey, hosted by the University’s research survey centre. Participants were excluded if their child was outside the age range (< 3 years or > 5 years) or if their child had been diagnosed with a neurodevelopmental or intellectual disorder. All measures (with the exception of demographic questions) required a response to prevent missing data. Participation was voluntary and anonymous, and participants were free to withdraw from the study at any time. Participants were first presented with an electronic copy of information and consent forms, and only those who provided digital consent went on to complete the online survey. Following survey completion, participants could choose to be directed to a separate survey to enter a prize draw for the chance to win $AU20–$50 gift cards and to provide student details to gain course credit for participation (if applicable).

## Phase 1: Item Development

The aims of Phase 1 were to generate items and assess the measure content through use of an expert panel. The item pool was designed to capture the six theoretically derived nighttime fear domains. The domains included sub-types of nighttime fear (including (1) personal safety fears, (2) separation fears, (3) imaginal/fantasy fears, and (4) inherent characteristics of the dark fears), as well as associated avoidance and interference behaviours, at (5) bedtime/sleep and (6) in the dark).

The hypothesised factor structure served as a framework for item creation [41] with items specifically generated to tap into each content area (i.e., factor). The instructions, factors and initial 62 item pool were generated based on a scientific literature review [1, 2, 4], as well as contribution from field professionals (three academics, two clinical psychologists, two PhD psychology candidates studying sleep and child anxiety, four provisional psychologists, two early primary school teachers, and a case manager from a large psychology clinic) and end users (two fathers and three mothers of preschoolers with nighttime fears).

It has been suggested that a minimum of 3 items, and preferably 5—6 items, are required to represent a factor [42]. As subsequent psychometric analyses were designed to reduce the final number of items, and as EFA performs better when factors are overdetermined, it was decided to be over inclusive (i.e., by a minimum of 50%) when generating items [41–43]. Therefore, for a proposed 6-factor solution with 5–7 items per factor as the desired scale size, between 48 to 66 items (8–11 per factor) was determined to be ideal. The response format used a 6-point Likert scale requiring parents to rate how true each item is of their child (0 = not at all true, 1 = rarely true, 2 = sometimes true, 3 = often true, 4 = very often true and 5 = always true), with reference to the previous week, or most recent regular week.

A neutral midpoint option was not provided in order to avoid complacency and indecisiveness. A greater number of options (i.e., 6) were included in order to increase precision and variability in measurement, as well as to increase internal consistency and dependability [40, 44, 45]. Items were written according to the basic principles described by Clark and Watson [43], Spruyt and Gozal [40], which highlight the importance of avoiding dated phrases, double-barrelled questions, complex wording and colloquialisms.

To examine the measure content, the scale was then sent for review to an expert panel from the USA and Australia (N = 7) with expertise in paediatric anxiety, sleep, and scale development. Experts were asked to provide feedback on clinical and research relevance, comprehensiveness, comprehensibility of individual items, the hypothesised factor structure, instructional blurb and rating scale. Following expert feedback, minor wording edits were made, and five items were removed.

## Phase 2: Scale Development

This phase aimed to pilot test the measure and conduct item reduction analysis before conducting an Exploratory Factor Analysis (EFA) to examine the factor structure.

### Pilot Test

The resulting pool of 57 items were then pilot tested with 120 parents of children aged 3 to 5 years. The aim of the pilot was to gain anecdotal feedback on the wording of the instructions and items, and to psychometrically examine the items for purposes of item reduction and refinement. Spruyt and Gozal [40] recommend that pilot trials are an important step before factor analysis to determine whether or not items, scale or layout need to be changed in any way (rather than simple deletion). The minimum N required to conduct a pilot test is generally the number of items on the scale plus one [41], with more than 100 participants being considered ideal [43]. Therefore, the 120 participants for the pilot test was deemed sufficient.

#### Pilot Test Participants

Detailed demographic information for each sample used in this research is outlined in Supplementary Table 1. The participants for the pilot test were 120 parents aged between 22 and 55 years (*M* = 36.10, *SD* = 5.47), who reported being either the mother (99.2%), or father (0.8%) of a child aged between 3 and 5 years old (*M* = 3.91, *SD* = 0.78). The majority of adult respondents were Caucasian (88.3%), married (79.2%) had a household income over AUD$100, 001 (67.5%), and had completed a bachelor degree (34.2%). The majority of children were male (59.2%) and lived with both parents (91.7%).

#### Pilot Test Item Reduction Analysis and Results

Items were considered for removal if they met both of the following criteria: (1) item redundancy or low correlations with other items (i.e., inter-item correlations of r > 0.80 or < 0.30 respectively); (2) poor item statistics (i.e., if all response options were not utilised, or there were corrected item-total correlations of r < 0.40). Using these criteria, the item pool was reduced to 37 items. There were no changes made to the wording of items, instructions or general layout based on participant feedback.

### Exploratory Factor Analysis

#### EFA Participants

The participants for the exploratory factor analysis (EFA) were 436 parents aged between 19 and 56 years (*M* = 33.88, *SD* = 6.65), who reported being either the mother (79.7%), father (14.5%), or primary caregiver (5.8%) of a child aged between 3 and 5 years old (*M* = 3.99, *SD* = 0.85). The majority of adult respondents were Caucasian (80.2%), married (64.1%) had a household income over AUD$100, 001 (45.8%), and had completed a bachelor degree (29.1%). Just over half of the children were female (50.2%) and the majority lived with both parents (76.6%). Refer to Supplementary Table 1 for further demographic details.

#### EFA Data Analysis

Prior to conducting the EFA, items were removed if they met two or more of the following criteria: (1) item redundancy or insufficient correlations with other items (i.e., inter-item correlations of r > 0.80 or < 0.30 respectively), (2) poor item statistics (i.e., if all response options were not utilised), or if there were corrected item-total correlations of r < 0.40, and/or (3) age bias (i.e., if a singular item correlated (r > 0.35) with the reported age of the child or parent [41, 43]. Eight items were excluded based on these criteria, leaving 29 items for the EFA, none of which required reverse scoring.

The R package ‘psych’ (v. 4.3.0) [46] was used to conduct a series of EFAs, using polychoric correlations and specifying principal axis factoring with oblique rotation (i.e., direct oblimin). This rotation technique was selected as factors were likely to be correlated [47]. The number of factors retained was established using parallel analysis [48], examination of the scree plot [49], the Kaiser–Guttman criterion (i.e., retention of factors with eigenvalues ≥ 1.0, [50, 51], and inspection of the pattern matrix. During factor extraction, items were assessed for poor primary-factor loading (i.e., < 0.40) or small communalities (i.e., < 0.40), cross-loading (i.e., secondary factor loadings of ≥ 0.30), lack of conceptual/face validity (i.e., loading of an item on a factor that did not align with theory or hypothesised factor), and whether or not they formed part of a non-robust factor (i.e., a factor with less than three items). Decisions regarding item exclusion and retention were both data- and theoretically driven [41, 43].

#### EFA Results

Barlett’s test of sphericity was significant (χ^2^ = 9711.95, df = 406, *p* < 0.001) and the Kaiser–Meyer–Olkin value was 0.96, indicating that the initial 29 items were appropriate for factor analysis. Additionally, all measures of sampling adequacy taken from the diagonal of the anti-image correlation table, were = > 0.80, and all assumptions were met. The scree plot, Kaiser–Guttman criterion, and parallel analysis converged on a four-factor solution, rather than the hypothesised 6-factor solution. Upon inspection of the pattern matrix, it was identified that the four types of nighttime fears (personal safety fears, separation fears, imaginal/fantasy fears, and inherent characteristics of the dark fears), collapsed onto a single factor, representing ‘Nighttime Fear Focus’. The second factor contained items pertaining to the avoidance and interference behaviours at bedtime/sleep representing ‘Bedtime/Sleep Avoidance and Interference’. The third factor pertained to both fear of the dark itself *and* the manifesting behaviours in the dark representing ‘Dark Fear’. The fourth factor consisted of three unrelated items that did not align with theory or the proposed factor structure and were consequently removed from the scale. Nine additional items were removed from the scale due to cross loading (5 items), misalignment with theory (one item), and redundancy (3 items).

A final EFA was conducted with the 17 remaining items, revealing a three-factor solution that explained 70% of the total variance. The final 17 items and scale statistics are presented in Table 1, with all items loading strongly onto their primary factor with no cross-loadings. The first factor (‘Nighttime Fear Focus’) consisted of eight items pertaining to various fears at nighttime (including personal safety fears, separation fears, imaginal/fantasy fears), with loadings ranging between 0.52 and 0.93. The second factor (‘Bedtime/Sleep Avoidance and Interference’) consisted of five items capturing bedtime/sleep avoidance behaviour and interference, with loadings between 0.63 and 0.96. The third factor (‘Dark Fear’) consisted of four items reflecting both fear of the dark and specific avoidance behaviour and interference of darkness, with loadings ranging between 0.75 and 0.87. The factors were moderately to strongly correlated with each other (r = 0.62–0.78).

**Table 1:** EFA and CFA factor loadings for the FAWN-YC items

		EFA				CFA		
		(N = 436)				(N = 383)		
		Factor LOADINGS				Standardized factor loadings		
	Item	NFF	BSAI	DF	h^2^	NFF	BSAI	DF
1	At nighttime my child has fears that someone is going to hurt them	0.93			0.77	0.66		
2	At nighttime my child worries about ghosts/spirits	0.87			0.73	0.65		
3	At nighttime my child is scared about their own safety, or the safety of loved ones	0.83			0.75	0.69		
4	At nighttime my child worries about scary animals	0.81			0.64	0.64		
5	At nighttime my child is afraid of having a nightmare/bad dream	0.70			0.66	0.69		
6	At nighttime my child worries about family members	0.69			0.60	0.53		
7	At nighttime my child worries about shadows in the room	0.57			0.68	0.76		
8	At nighttime my child worries about banging or knocking noises	0.52			0.60	0.73		
9	Because of fears at night, my child does not want to go to bed		0.96		0.82		0.87	
10	Because of fears at night, my child cries at bedtime		0.89		0.85		0.84	
11	Because of fears at night, my child tantrums or argues with parent(s) or others at bedtime		0.80		0.69		0.79	
12	Because of fears at night, my child calls out after bedtime		0.74		0.73		0.80	
13	My sleep/other family member’s sleep is disrupted due their nighttime fears		0.63		0.59		0.76	
14	My child must have a bright light on to walk into a room			0.87	0.76			0.77
15	My child is frightened of the dark			0.86	0.83			0.90
16	Because of fears at night, my child is unable to sleep in total darkness			0.77	0.56			0.63
17	My child is fearful of going into dark places			0.75	0.71			0.86
	Item variance explained %	30	22	18				
	Cronbach’s α	0.92	0.90	0.88		0.87	0.90	0.86
	Mean	9.47	7.18	7.62		10.91	8.85	9.91
	Standard deviation	8.01	5.68	5.28		7.73	6.54	5.72

*NFF* nighttime fear focus, *BSAI* bedtime/sleep avoidance and interference, *DF* dark fear, *h*^2^ communalities

## Phase 3: Scale Evaluation

Phase 3 aimed to confirm the factor structure of the 17-item FAWN-YC, examine the possibility of a general factor structure and examine the psychometric properties of validity (convergent validity and divergent validity) and reliability (internal consistency and test–retest reliability over a 2-week period).

### Phase 3 Procedure and Participants

The same recruitment methods were used as those outlined in the General Methods except that, at the end of the survey, participants were given the opportunity to provide their unique participant identification code and email address in order to be contacted to complete an additional, later survey for the purpose of test–retest reliability.

The participants were 383 parents and primary caregivers aged between 21 and 66 years (*M* = 35.39, *SD* = 6.11), who reported being either the mother (96.3%), father (2.1%) or primary caregiver (0.5%) of a child aged between 3 and 5 years old (*M* = 4.33, *SD* = 0.79). Fifteen participants began the questionnaire but were excluded due to having children with neurodevelopmental disorders, and another 5 were excluded due to having children outside the selected age range. The majority of adult respondents were Caucasian (90%), married (65%) had a household income over AUD$100, 001 (57.2%), and had completed a bachelor degree (34.5%). Just over half of the children were male (52.0%) and the majority lived with both parents (79.4%). Refer to Supplementary Table 1 for further demographic details.

### Confirmatory Factor Analysis

#### CFA Data Analysis

Prior to analysis, descriptive statistics were examined for outliers, and assumptions were all checked and met. The data were analysed using IBM SPSS Statistics (Version 29) analytic software and Amos Graphics (Version 29). Amos Graphics was used to conduct a CFA with a robust maximum likelihood estimation to confirm the factor structure identified in the EFA. To evaluate model fit, several commonly used indices were considered (χ^2^, χ^2^ relative to sample size, adjusted goodness of fit index (AGFI), comparative fit index (CFI), and root mean square error of association (RMSEA)). While a non-significant χ^2^ is indicative of “good” model fit, it is sensitive to large sample sizes, such as that used in the current study. For a large sample size, it is therefore recommended to divide χ^2^ by the degrees of freedom with ratios of 2–3 indicative of “good” model fit [52]. Adequate model fit can also be determined by an AGFI greater than 0.90 and a CFI greater than 0.95. A RMSEA less than 0.05 is also considered “good” model fit, values between 0.05 and 0.08 indicative of “fair” and “acceptable” fit, and values between 0.08 and 0.10 indicative of “mediocre fit” [53]. The model was also compared to a general-factor model (i.e., whereby all 17 items were allowed to load onto a single latent factor) using the Akaike Information Criterion (AIC). Lower AIC values indicate a model that is more parsimonious and better-fitting [54].

#### CFA Results

A CFA was performed with items constrained to load onto their respective factors, and factors allowed to covary as per the EFA results and a-priori theory. According to model fit indices, the hypothesised measurement model had acceptable to good fit to the data in this sample, *χ*^2^ (114, *N* = 383) = 275.51, *p* < 0.001, *χ*^2^/*df* = 2.42, AGFI = 0.90, CFI = 0.96, RMSEA = 0.06 (CI90 = 0.05, 0.07) and AIC = 353.51 and explained a total of 70% of the variance. The standardised confirmatory factor loadings for each item are presented in Table 1. The Cronbach alphas for each subscale (α = 0.86–0.90) and the total score (α = 0.92) were acceptable, with moderate to strong correlations between each subscale (r = 0.54–0.83).

A general factor model was also examined, whereby all 17 items were allowed to load onto a single latent factor. This model had poor fit, χ^2^ (119, N = 383) = 1228.00, p < 0.001, χ^2^/df = 10.319, AGFI = 0.55, CFI = 0.70, RMSEA = 0.16 (CI90 = 0.15, 0.16) and AIC = 1296.00 While the AIC revealed that the general factor model had poorer fit compared to the measurement model, the standardized loadings for each item in the general factor model were moderate to strong (0.55 to 0.75). There were also strong positive correlations found between the total score and the three subscales and furthermore, the total score demonstrated acceptable internal consistency in both EFA and CFA (α = 0.95 and 0.92 respectively). Therefore, while model fit suggests the 3-factor model is a considerably better fit that the general factor model, the strength in the other psychometric properties provide preliminary evidence for use of a total FAWN-YC score.

### Psychometric Tests of Validity and Reliability

IBM SPSS Statistics (Version 29) analytic software was used to conduct all tests of validity and reliability.

### Validity: Convergent and Divergent Validity

#### Convergent and Divergent Validity Measures

##### Sleep Problems

The total score and Sleep Anxiety subscale score of the Children’s Sleep Habits Questionnaire (CSHQ; [28] were used to examine the convergent validity of the FAWN-YC. The CSHQ is a 33-item (e.g. “*Child needs parent in the room to fall asleep*”) parent-report instrument that contains items related to common sleep behaviours in children. Items are rated on a three-point Likert scale (1 = rarely [0–1 night per week] to 3 = usually [5–7 nights per week]), with each question asked in relation to the previous week. The total score, calculated by summing all items, may range from 33 to 99, with higher scores indicating more problematic child sleep behaviours, and total scores over 41 being indicative of a clinical level paediatric sleep problem [28]. The 4-item Sleep Anxiety subscale is calculated by summing the 4 component items, and may range from 4 to 12, with higher scores indicative of greater sleep anxiety.

The CSHQ has been used with parents of children from early childhood to early adolescence [33] and has shown acceptable total internal consistency with both community (α = 0.72; [55]) and clinical (α = 0.77; [29, 30]) samples of parents of young children aged 3 to 5 years. The CSHQ total score has also demonstrated acceptable test–retest reliability (range between 0.62 and 0.79 [28]). The Sleep Anxiety subscale has demonstrated slightly lower than acceptable internal consistency with both community (0.63) and clinical (0.68) samples [28].

##### Anxiety

The Preschool Anxiety Scale (PAS; [56]) measures child anxiety and was used to examine convergent validity. The PAS is a 28-item (e.g. “*Is tense, restless or irritable due to worrying*”) parent-report instrument designed for children aged 3 to 5 years. The items are rated on a 5-point scale from 0 = *not at all true* to 4 = *very often true*. Items are summed to produce a total score that may range from 0 to 112, with higher scores indicating greater anxiety. The PAS has a strong evidence base, including evidence of convergent and divergent validity in clinical and community populations, and evidence of sensitivity to intervention effects and strong inter-assessor agreement [13]. Internal consistency in previous studies of preschoolers using the total PAS has been acceptable (α = 0.92; [30]).

##### Mythical Creatures Fears and Vulnerability Fears

The 13-item Mythical Creatures Fears (e.g. ghosts or spooky things) and 7-item Vulnerability Fears (e.g. being alone) subscales of The Modified Fear Survey Schedule for Children–II (FSSC– IIP; [57]) were used to examine convergent validity. The FSSC– IIP is a parent-report survey specifically designed for parents of children aged 3 to 9 years, with parents asked to rate their child’s level of fear of the subject of each item (e.g. imaginary creatures) on a 3-point scale from 1 = *not scared (not applicable)* to 3 = *very scared*. Items on the subscale are summed to produce a subscale score, with higher scores indicating greater fear of mythical (imaginary) creatures or the feeling of vulnerability (being alone, being in the dark). The factor structure of the FSSC– IIP has been supported in a large Australian sample [57] and acceptable internal consistency has been demonstrated (α = 0.70; [58]).

##### Conduct Problems, Emotional Symptoms and Prosocial Behaviours

The Conduct Problems and Emotional Symptoms subscales of the Strengths and Difficulties Questionnaire (SDQ; [59]) were used to examine the convergent validity, while the Prosocial Behaviours subscale was used to measure divergent validity. The SDQ is a 25-item parent-report instrument for use with parents of children aged 2–17 years. Items on the SDQ are rated on a three-point Likert scale (0 = not true to 2 = certainly true) with each question asked in relation to the previous 6-month period. Higher scores on the Conduct Problems and Emotional Symptoms subscales are indicative of greater problems, while higher scores on the Prosocial Behaviour subscale indicate more positive behaviours. The SDQ has shown satisfactory construct validity and acceptable internal consistency with large samples of parents of young children aged 3 to 5 years (Conduct Problems, Emotional Problems and Prosocial Behaviour subscale α ranges of mothers and fathers = 0.72 to 0.84 [60]).

#### Convergent and Divergent Validity Data Analysis and Results

Prior to analysis, descriptive statistics were examined for outliers, and assumptions were all checked and met. Bivariate correlations were used to assess convergent and divergent validity. Table 2 presents the means, standard deviations, bivariate correlations and Cronbach’s alphas of all variables used in this phase. Correlations between the FAWN-YC composite and subscale scores and measure of convergent validity were all significant and in the predicted directions (*r* = 0.30 to 0.82, *p* < 0.01). With respect to divergent validity, correlations between the FAWN-YC composite score and subscales and the SDQ prosocial subscale were all non-significant, with the exception of a very weak negative correlation between the Dark Fear subscale and the prosocial subscale of the SDQ (*r* = − 0.10, *p* < 0.05).

**Table 2:** Correlations between the FAWN-YC and selected measures of convergent and discriminant validity

		1	2	3	4	5	6	7	8	9	10	11	12
1	FAWN-YC Total	1											
2	FAWN-YC NFF	0.88**	1										
3	FAWN-YC BSAI	0.83**	0.57**	1									
4	FAWN-YC DF	0.81**	0.58**	0.54**	1								
5	CSHQ Total	0.67**	0.53**	0.73**	0.44**	1							
6	CSHQ SA	0.61**	0.43**	0.59**	0.55**	0.70**	1						
7	PAS	0.73**	0.72**	0.56**	0.55**	0.54**	0.47**	1					
8	FSSC-IIP MC	0.50**	0.58**	0.31**	0.34**	0.32**	0.23**	0.41**	1				
9	FSSC-IIP VF	0.82**	0.67**	0.64**	0.79**	0.55**	0.59**	0.67**	0.46**	1			
10	SDQ-C	0.41**	0.35**	0.39**	0.30**	0.39**	0.23**	0.29**	0.20**	0.35**	1		
11	SDQ-E	0.62**	0.59**	0.51**	0.47**	0.48**	0.40**	0.76**	0.34**	0.61**	0.42**	1	
12	SDQ-P	− 0.11	− 0.07	− 0.10	− 0.10*	− 0.17**	− 0.08	− 0.13*	− 0.08	− 0.02	− 0.40**	− 0.19**	1
	Cronbach’s Alpha	0.92	0.87	0.90	0.86	0.86	0.65	0.92	0.90	0.84	0.69	0.75	0.77
	Mean	29.57	10.91	8.85	9.81	48.17	7.27	28.91	16.20	11.85	2.36	2.59	7.29
	Standard Deviation	16.87	7.73	6.54	5.72	9.56	2.30	18.09	4.23	3.41	1.87	2.28	2.17

FAWN-YC total = composite score, FAWN-YC NFF = night-time fear focus, FAWN-YC BSAI = bedtime/sleep avoidance and interference, FAWN-YC DF = dark fear, CSHQ Total = child sleep habits questionnaire, CSHQ SA = sleep anxiety, PAS = preschool anxiety scale, FSSC-IIP MC = the Modified fear survey schedule for children–II mythical creatures fears, FSSC-IIP VF = vulnerability fears, SDQ-C strengths and difficulties questionnaire conduct problems, SDQ-E = emotional symptoms, SDQ-P = prosocial behaviours

*p < 0.05, **p < 0.01

### Reliability: Internal Consistency

Cronbach’s α was used to assess the internal consistency of the FAWN-YC factors in both the EFA and CFA samples, which are reported in Table 1. As cited in Godfred et al. [39], when it comes to validating scales α = 0.70 is acceptable, and between α = 0.80 and 0.95 is preferred. In both the EFA and CFA samples, the total score reached preferred internal consistency in (α = 0.95 and 0.92 respectively). The internal consistency of each factor also reached preferred reliability (Cronbach’s α = 0.86–0.92).

### Reliability: Test–Retest

#### Test–Retest Reliability Procedure and Participants

Participants from phase 3 recruitment who consented to be contacted again were invited via email to complete the FAWN-YC two weeks after the first administration. Responses recorded at Time 1 and Time 2 were matched using a unique participant identification code. Only participants who completed the second assessment within the two-week retest period were included. Of the 383 participants, 229 consented to be contacted and entered their unique code and email address. Of the 229, 120 were contactable and were emailed a link to complete the survey within the allocated time frame (i.e., ± 48 h from 2 weeks post completion of the phase 3 initial survey). In the email, participants were reminded of their unique code and given instructions to enter it at the beginning of the survey. Of the 120 participants contacted, 52 went on to complete the retest survey within the allocated time period. The resulting 52 participants were aged between 21 and 47 years (M = 34.46, *SD* = 5.85) and reported being either the mother (98.1%) or father (1.9%) of a child aged between 3 and 5 years old (M = 4.92, *SD* = 1.19). The majority of adult respondents were Caucasian (84.6%), married (63.5%) and had completed a bachelor degree (59.6%), with just over half of the families having a household income over AUD$100, 001 (50%), The majority of children were male (57.7%) and living with both parents (80.8%).

#### Test–Retest Data Analysis and Results

Intraclass correlation coefficient estimates, 95% confidence intervals based on a 2-way mixed-effects model with absolute-agreement were run. Confidence interval values greater than 0.90 indicate excellent reliability, values between 0.75 and 0.9 suggest good reliability, values between 0.5 and 0.75 suggest moderate reliability and values less than 0.5 suggest poor test–retest reliability [61]. Intraclass correlation coefficient estimates suggested temporal stability over a two-week period for the FAWN-YC total score and subscales. The FAWN-YC composite score was considered excellent (ICC = 0.95, 95% CI [0.90, 0.97]), as was the Dark Fears subscale (ICC = 0.95, 95% CI [0.92, 0.97]). Both the Nighttime Fear Focus subscale (ICC = 0.88, 95% CI [0.79, 0.93]) and the Bedtime/Sleep Avoidance and Interference subscale (ICC = 0.93, 95% CI [0.86, 0.96]) were considered to have good reliability.

## Discussion

The purpose of this research was to develop and psychometrically evaluate the Fears and Worries at Nighttime—Young Children (FAWN-YC); a parent-rated measure for children aged 3–5 years (freely available in the Supplementary materials including scoring key). Phase 1 aimed to generate items and assess the measure content through use of an expert panel. Phase 2 aimed to pilot test the measure, conduct item reduction analysis and conduct an Exploratory Factor Analysis (EFA) to examine the factor structure. Phase 3 aimed to confirm the factor structure using Confirmatory Factor Analysis (CFA) and examine the psychometric properties of validity (convergent and divergent) and reliability (internal consistency and test–retest). Although 6 subscales were originally proposed, the results of Phases 2 and 3 indicated a 3-factor structure best fit the data. The final 17 item scale consisted of three subscales measuring: Nighttime Fear Focus (8 items), Bedtime/Sleep Avoidance and Interference (5 items) and Dark Fear including avoidance and interference of darkness (4 items). The internal consistency of the three subscales and total score was found to be acceptable, and the convergent and divergent validity of the scale was supported. Finally, the FAWN-YC total score and subscales demonstrated test–retest reliability, indicating temporal stability over a two-week period.

The total score and subscales of the FAWN-YC demonstrated strong psychometrics including internal consistency, test–retest reliability, convergent validity and divergent validity suggesting that the scale is a psychometrically sound and valid measure of nighttime fears and worries in young children, that will provide a useful instrument for researchers and clinicians alike. Indeed, although our findings support the independent use of the three FAWN-YC subscales over the composite score, the total score demonstrated very strong psychometric properties, providing preliminary support for its use.

With regards to convergent validity, Phase 3 demonstrated that the FAWN-YC total score and its three subscales were all significantly and positively correlated with theoretically and empirically linked variables related to nighttime fears in young children, with the strength of each of these correlations making theoretical sense. For instance, the ‘Nighttime Fear Focus’ subscale correlated most strongly with a measure of child anxiety (PAS; [56]), the ‘Bedtime/Sleep Avoidance and Interference’ subscale correlated most strongly with a measure of total child sleep problems (CSHQ [28]), and the ‘Dark Fear’ subscale correlated most strongly with the Vulnerabilities subscale of the Modified Fear Survey Schedule for Children–II (FSSC-IIP [57]), which has three (of seven) items related directly to fear of the dark. Overall, the findings suggest that higher levels of nighttime and darkness fears and their behavioural manifestations are related to higher levels of both internalising (e.g., anxiety), and externalising (e.g., conduct problems) behaviours, as well as sleep problems. The findings therefore are consistent with previous research reporting links between nighttime fears, internalising, externalising, and sleep problems in preschool aged children [8, 9] and thus support the overall validity of the subscale and total scale scores.

Although psychometrically strong, the factor structure of the FAWN-YC is different to what was predicted. Six factors were originally hypothesised, with four factors predicted to cluster around distinct fear categories (personal safety fears, separation fears, imaginal/fantasy fears, inherent characteristics of the dark fears) and two representing different types of interference (bedtime/sleep time and the dark/night). However, while the ‘Bedtime/Sleep Avoidance and Interference’ factor emerged as distinct, the different types of nighttime fears and the dark avoidance and interference factors did *not*, and instead emerged as two factors. Most nighttime fear types clustered onto the *one* factor, ‘Nighttime Fear Focus’, which was characterised by worries and fear of things at nighttime (being alone, safety, imaginal), whereas fear of dark itself *and* avoidance and interference in the dark merged together to create a separate factor (i.e., ‘Dark Fear’). Given the way in which these factors have emerged, combined with evidence of correlation strengths and convergent validity, children who score high in the ‘Nighttime Fear Focus’ factor may be more representative of children with anxiety (i.e., separation anxiety, generalised anxiety), whereas children scoring high in ‘Dark Fear’ may represent more fear-based symptomology (i.e., specific phobia of the dark). Future research could examine whether this measure could predict distinct comorbidity clusters using clinical samples.

Although the predicted factor structure was based on a review of the literature [1, 2, 4], the literature reviewed was less than optimal in several ways. First, most studies examining nighttime fear types in children have focused on primary school aged children and young adolescents, rarely including preschool children. Given the specific developmental characteristics of preschool children, it may be that younger children experience fears at night differently to their older counterparts being more likely to experience a range of nighttime fears rather than specific ones. Second, only one study (that did not include children under 8.5 years of age, that used a relatively small sample from the Netherlands, and that was conducted almost 4 decades ago) examined the factor structure of their reported measure [3]. All other studies either clustered fears based on face validity or based their categories on the one study that did use factor analysis [1, 2, 4]. Although this research requires replication, the findings regarding factor structure speak to the importance of developing a nighttime fear measure specifically for preschool children, and highlight the differences between young children and their older child and teenage counterparts.

It is noteworthy, and indeed surprising, that items related to co-sleeping were ‘dropped’ from the ‘Bedtime/Sleep Avoidance and Interference’ factor due to poor primary-factor loading, insufficient correlations with other items and poor item statistics. When examining the behavioural manifestations of nighttime fears, the authors chose to use the sentence starter “Because of fears at nighttime, my child…” with the intention to gather information pertaining to parental perception of the behaviours directly related to child nighttime fears. The fact that co-sleeping items were dropped from this subscale therefore suggests that reactive co-sleeping may *not* be the result of nighttime fears in preschool aged children. Interestingly, a recent systematic review and cross-cultural meta-analysis examining co-sleeping and sleep problems in childhood noted the inconsistency in the relationship between co-sleeping and sleep anxiety and concluded that there is insufficient evidence to suggest a causal relationship [62]. Furthermore, most of the studies included in the review by Peng et al. did not include children in the preschool developmental period, with most co-sleeping literature focussed either on infants or primary school aged children. It may well be that co-sleeping is better explained by other child and parent factors rather than nighttime fears. The factors may possibly include child anxiety disorders (particularly separation anxiety) [63, 64], child behavioural difficulties such as bedtime resistance) [62] and even parental distress [23, 65]. Clearly reactive co-sleeping in the preschool development period warrants further investigation in this regard.

## Limitations and Directions for Future Research

This series of research allowed for the systematic development and psychometric testing of the FAWN-YC, using large sample sizes, and advanced statistical techniques. However, there were a number of limitations that should be noted. First, sensitivity and specificity were not tested, nor was this sample large enough, or demographically heterogenous enough, to determine norms. Collecting a larger, more diverse sample in the future, and including an accompanying clinical interview, would allow examination of the ability of the FAWN-YC to differentiate between children with and without problematic nighttime fears, and to determine norms. Second, the generalisability of the results of this study was limited by the proportion of male to female parents and caregivers completing the surveys. Although large sample studies indicate good inter-parent agreement in ratings of behavioural and emotional problems in preschool-aged children [66], future research would benefit from further attempts to recruit fathers in parent samples. Third, the sample was homogenous in terms of ethnicity, level of education, and wealth, thus limiting the generalisability of results to other populations. Future studies should strive to include samples that are more heterogenous in terms of these constructs. Finally, previous research suggests that parents can underestimate the frequency and intensity of their young child’s fears [67]. As is the case for all measures of anxiety and internal thoughts and states in the preschool developmental period, the FAWN-YC total score, and in particular the Nighttime Fear Focus subscale, may be susceptible to variance in the child’s ability to understand and share their fears and worries. It may also be susceptible to variance due to the parent’s ability to perceive their child’s fears and worries, which is indeed an important area for future inquiry. Similarly, as young children may lack the cognitive sophistication to respond to questionnaires and interviews, future research may include more objective measures (i.e., skin conductance, actigraphy or recordings/observations of child’s nighttime behaviours) to further validate the FAWN-YC.

## Implications and Conclusions

The findings of this research indicate that the 17-item Fears and Worries at Nighttime—Young Children (FAWN-YC) is a psychometrically valid, parent-report scale of nighttime fears. The FAWN-YC provides researchers and clinicians with a valid and reliable tool to assess the specific nighttime fear the child may have, the variety of nighttime fears the child has, and importantly, parental perceptions regarding how nighttime fears are affecting their child’s nighttime behaviours.

For researchers, The FAWN-YC provides a user-friendly, psychometrically valid measure of nighttime fears in children that may be used in a variety of studies including epidemiological studies, studies examining the antecedents and consequences of nighttime fears, and studies aiming to determine the efficacy of treatment programs designed to treat nighttime fears in this population.

For clinicians, the FAWN-YC may assist in the conceptualisation and treatment of children with nighttime fears. For example, although the focus of the fears/worries did not cluster into separate categories as expected (e.g., separation, imaginal, etc.), clinicians may still examine the scale at an item level to identify specific target fears in treatment. Thus, the brevity of the scale provides an efficient means of gathering information from parents to use for case formulation and treatment planning. Indeed, clinicians may use subscale scores to determine whether the child’s fears and worries are interfering with sleep and bedtime behaviours (subscale level) and in what specific manner (item level), or if the child is also afraid of the dark (subscale level).Therefore, the FAWN-YC may assist in identifying the target of an exposure hierarchy and the requirement for combinations of child relaxation skills, co-regulation skills, and/or parent upskilling such as behaviour management, exposure games and psychoeducation. Thus, the FAWN-YC is likely to be of significant value in both research and clinical settings, informing our understanding and treatment of fear, anxiety and sleep difficulties in preschool aged children.

## Summary

While childhood nighttime fears can be developmentally normal and transient in nature, approximately 10 to 30% of young children experience fear at night that is severe, persistent, interferes substantially with sleep, and requires significant family accommodation [4–7]. While literature examining nighttime fears in the preschool developmental period (3–5 years) is expanding, a validated measure of nighttime fears in young children does not yet exist. This paper outlines the development and psychometric evaluation of the Fears and Worries at Nighttime—Young Children (FAWN-YC) scale, a parent-rated measure for children aged 3–5 years. Based on previous literature, it was hypothesised that the measure would be represented by a six-factor solution, with four clusters of fear types (separation fears, darkness-related fears, personal safety fears and fears of the imagination) and two behavioural manifestations of fears (behaviours at bedtime/sleep and behaviours in the dark). However, exploratory factor analysis (EFA; N = 436) and confirmatory factor analysis (CFA; N = 383), resulted in a final 17 items that loaded onto only 3 factors: Nighttime Fear Focus (8 items, α = 0.92), Bedtime/Sleep Avoidance and Interference (5 items, α = 0.90), and Dark Fear (4 items, α = 0.88). The findings of this factor structure speak to the importance of developing this measure specifically for this unique developmental period, and highlights the differences between preschoolers and their older child and teenage counterparts. Evidence of convergent validity was found through strong associations between the total score and subscales of the FAWN-YC with measures of child anxiety, fear, sleep, externalising and conduct problems. Furthermore, there was support for divergent validity, and evidence for temporal stability over a 2-week period. Overall, the results provide strong preliminary evidence for the reliability and validity of the FAWN-YC total score and subscales and evidence for the importance of developing a nighttime fear measure specifically for preschool aged children. The FAWN-YC provides a psychometrically valid and user-friendly measure of nighttime fears in young children. This measure is likely to be of significant value in both research and clinical settings, informing our understanding and treatment of fear, anxiety and sleep difficulties in preschool aged children.

## Supplementary Information

Below is the link to the electronic supplementary material.

Supplementary file2 (DOCX 21 KB)

Supplementary file1 (PDF 121 KB)

## Data Availability

Please contact the corresponding authors of this paper to request access to data.
