# Supplementary material for: Fears and Worries at Nighttime in Young Children: Development and Psychometric Validation of a Parent-Report Measure (FAWN-YC)
Source: Child Psychiatry Hum Dev. 2024 Sep 16;57(3):941–53. doi: 10.1007/s10578-024-01758-3 (PMC13201348; doi:10.1007/s10578-024-01758-3)
Supplement: Supplementary file 1 — Supplementary file2 (DOCX 21 KB) [file 10578_2024_1758_MOESM1_ESM.docx]

**Supplementary Table 1**

*Demographic Characteristics and Descriptive Statistics for Participants in Each Sample*

|  | Pilot Study  N = 120 | | EFA Sample  N = 436 | | CFA Sample  N = 383 | |
| --- | --- | --- | --- | --- | --- | --- |
| Demographic Characteristics | M (SD) | % | M (SD) | % | M (SD) | % |
| Parent Age (years) | 36.10 (5.74) |  | 33.88 (6.65) |  | 35.39 (6.11) |  |
| Female (%) |  | 99.2 |  | 82.6 |  | 94.5 |
| Male (%) |  | 0.8 |  | 17.4 |  | 5.0 |
| Child Age (years) | 3.91 (0.78) |  | 3.99 (0.85) |  | 4.33 (0.79) |  |
| Female (%) |  | 40.8 |  | 50.2 |  | 47.8 |
| Male (%) |  | 59.2 |  | 49.8 |  | 52.0 |
| Household income (AUD) |  |  |  |  |  |  |
| < $60,000 |  | 8.3 |  | 22.7 |  | 18.3 |
| $60,001 - $80,000 |  | 12.5 |  | 14.9 |  | 6.5 |
| $80,001 - $100,000 |  | 11.7 |  | 15.6 |  | 13.3 |
| $100,001 - $200,000 |  | 45.0 |  | 31.2 |  | 47.3 |
| More than $200,000 |  | 16.7 |  | 14.6 |  | 9.9 |
| No response |  | 5.8 |  | 0 |  | 4.7 |
| Parent Highest Level of Education | | | | | | |
| Below grade 12 |  | 3.3 |  | 4.9 |  | 8.0 |
| Grade 12 |  | 12.5 |  | 19.5 |  | 11.7 |
| Associate or technical degree (TAFE) |  | 18.3 |  | 28.0 |  | 23.2 |
| Bachelor degree |  | 34.2 |  | 29.1 |  | 34.5 |
| Postgraduate degree |  | 31.7 |  | 18.6 |  | 22.5 |
| Parent Ethnicity |  |  |  |  |  |  |
| Caucasian |  | 88.3 |  | 80.2 |  | 90.0 |
| Asian |  | 7.5 |  | 7.2 |  | 4.3 |
| Aboriginal or Torres Strait Islander |  | 1.7 |  | 5.3 |  | 4.1 |
| Pacific Islander |  | 0 |  | 3.1 |  | 0.8 |
| Black/African |  | 0 |  | 2.4 |  | 0.3 |
| Other or Unknown |  | 2.5 |  | 0 |  | .5 |
| Child Ethnicity |  |  |  |  |  |  |
| Caucasian |  | 87.5 |  | 80.9 |  | 90.1 |
| Asian |  | 4.2 |  | 7.2 |  | 2.8 |
| Aboriginal or Torres Strait Islander |  | 1.7 |  | 5.5 |  | 5.7 |
| Pacific Islander |  | 0 |  | 2.0 |  | 0.6 |
| Black/African |  | 0 |  | 3.0 |  | 0.3 |
| Other or Unknown |  | 6.7 |  | 1.4 |  | 0.6 |
| Employment Status |  |  |  |  |  |  |
| Student |  | 15.0 |  | 20.6 |  | 9.4 |
| Employed full-time |  | 25.0 |  | 38.3 |  | 31.1 |
| Employed part-time |  | 48.3 |  | 33.5 |  | 39.9 |
| Unemployed |  | 10.8 |  | 6.9 |  | 16.4 |
| Other |  | .8 |  | .7 |  | 3.1 |
| Marital Status |  |  |  |  |  |  |
| Never married or single |  | 4.1 |  | 10.3 |  | 10.7 |
| Dating/Defacto* |  | 14.2 |  | 20.9 |  | 20.8 |
| Married |  | 79.2 |  | 64.1 |  | 65.0 |
| Divorced/Separated |  | 2.5 |  | 4.6 |  | 3.3 |
| Child Living Arrangement |  |  |  |  |  |  |
| Living with both parents |  | 91.7 |  | 76.6 |  | 79.4 |
| Living with mother only |  | 4.2 |  | 13.7 |  | 12.1 |
| Living with father only |  | 0 |  | 2.3 |  | 0.3 |
| Living with parent and new partner |  | 3.3 |  | 7.4 |  | 5.9 |
| Other |  | 0.8 |  | 0 |  | 0.3 |

**Note. Defacto refers to an unmarried couple living together*
