# Supplementary material for: Fears and Worries at Nighttime in Young Children: Development and Psychometric Validation of a Parent-Report Measure (FAWN-YC)
Source: Child Psychiatry Hum Dev. 2024 Sep 16;57(3):941–53. doi: 10.1007/s10578-024-01758-3 (PMC13201348; doi:10.1007/s10578-024-01758-3)
Supplement: Supplementary file 2 — Supplementary file1 (PDF 121 KB) [file 10578_2024_1758_MOESM2_ESM.pdf]

Fears And Worries at Nighttime – Young Child (FAWN-YC) **ID:** ________________________________ **Date:** ____________

The following statements are about child behaviours at **nighttime**. Please rate how true the statements are for your child at nighttime over the past week. If the last week was unusual (e.g. on holidays or your child was unwell), answer the questions based on a typical week. It may be distressing to think about whether your child has these thoughts or shows these behaviours, but it is important to carefully answer each question as accurately as you can.

**At nighttime my child…**	Not at all true N/A	Rarely true	Sometimes true	Often true	Very often true	Always true	Admin Use
has fears that someone is going to hurt them	0	1	2	3	4	5	
worries about ghosts / spirits	0	1	2	3	4	5	
is scared about their own safety, or the safety of loved one	0	1	2	3	4	5	
worries about scary animals	0	1	2	3	4	5	
is afraid of having a nightmare / bad dream	0	1	2	3	4	5	
worries about family members	0	1	2	3	4	5	
worries about shadows in the room	0	1	2	3	4	5	
worries about banging or knocking noises	0	1	2	3	4	5	
**Because of fears at nighttime my child…**							
does not want to go to bed	0	1	2	3	4	5	
cries at bedtime	0	1	2	3	4	5	
tantrums or argues with parent(s) or others at bedtime	0	1	2	3	4	5	
calls out after bedtime	0	1	2	3	4	5	
my sleep/other family member's sleep is disrupted	0	1	2	3	4	5	
is unable to sleep in total darkness	0	1	2	3	4	5	
**My Child…**							
must have a bright light on to walk into a room	0	1	2	3	4	5	
is frightened of the dark	0	1	2	3	4	5	
is fearful of going into dark places	0	1	2	3	4	5	

NFF	BSAI	DF

☐ **Nighttime Fear Focus**
☐ **Bedtime/Sleep Avoidance and Interference**
☐ **Dark Fears**
☐ **Total Score**
